# Localizing health emergency preparedness and response: emergency medical team development and operations in Pacific island countries and areas

**DOI:** 10.5365/wpsar.2023.14.6.1021

**Published:** 2023-06-15

**Authors:** Sean T Casey, Erin Noste, Anthony T Cook, Jan-Erik Larsen, Simon Cowie, May M Ferguson, Pierre-Yves Beauchemin

**Affiliations:** a World Health Organization Regional Office for the Western Pacific, Manila, Philippines.; b School of Population Health, University of New South Wales, Sydney, New South Wales, Australia.; c Department of Emergency Medicine, University of California San Diego, California, United States of America.

Since 2010, the World Health Organization (WHO) has worked with Member States and nongovernmental organizations around the world through its Emergency Medical Team (EMT) Initiative to build a network of deployable clinical rapid response teams. Capable of national and/or international response, EMTs apply common principles and minimum standards as detailed in WHO’s *Classification and Minimum Standards for Emergency Medical Teams* (2021), also known as the “Blue Book” (originally published in 2013 as *Classification and Minimum Standards for Foreign Medical Teams in Sudden Onset Disasters*). (*1*)

In the Pacific, island nations face threats from emerging infectious diseases, natural hazards and the long-term impacts of climate change. Recognition of these threats has led to increased investments and focus on health emergency preparedness, readiness, response, recovery and resilience. In response to the impacts of many tropical cyclones and typhoons, recent volcanic eruptions and tsunamis, measles outbreaks in 2019, and the lingering effects of the coronavirus disease (COVID-19) pandemic across the region, Pacific governments have increased investment in national response capacities, including by establishing and activating deployable, self-sufficient national EMTs (**Fig. 1**). (*2*-*8*)

**Fig. 1:**
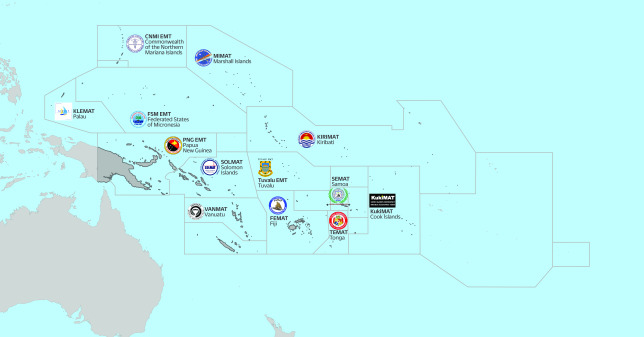
Pacific emergency medical teams established and in development

[**insert** Figure 1]

## EMERGENCY MEDICAL TEAM DEVELOPMENT IN THE PACIFIC

In the first years of EMT development in the Pacific, national teams were established in four countries: Fiji, Solomon Islands, Tonga and Vanuatu. Support was provided by WHO with funding and technical support from the governments of Australia and New Zealand to: (a) train Pacific EMT members; (b) provide a “cache” of EMT equipment and supplies for self-sufficient deployment; (c) develop national EMT standard operating procedures (SOPs) in each country; and (d) formalize and ensure national ownership of each EMT. (*2*) Pacific EMT development support and training is based on WHO’s Blue Book, with necessary adaptations for small Pacific island contexts, which have smaller human resource pools, infrastructure and storage limitations, financial constraints and extremely challenging logistics. (*9*-*11*)

Between 2017 and 2019, before the COVID-19 pandemic and extensive border closures across the Pacific region, 5-day in-person training workshops with full-scale simulation exercises were held in Fiji, Solomon Islands, Tonga and Vanuatu. EMT mentors with extensive experience in developing EMTs and deploying as team members provided both remote and on-site support while Pacific teams formalized their structures and developed their national SOPs. At the same time, Pacific EMTs recruited team members from within Pacific ministries of health and other government agencies (including police and fire services) to form national EMT member rosters.

Through procurements, donations and leveraging existing in-country clinical and non-clinical equipment and supplies, Pacific EMTs became self-sufficient and equipped to deploy to outbreaks and disasters in remote and austere conditions without burdening local resources. EMTs established standalone field operations, ensured safe food and water for patients and staff, and provided clinical care according to national standards in response to emergencies within their own borders. (*6*-*8*)

Subsequently, building on the success of national EMT development in several countries in the South Pacific, additional Pacific island countries and areas (PICs) committed to establishing their own national EMTs. Following the same model, teams were established in the Cook Islands and in the Commonwealth of the Northern Mariana Islands in 2019. (*3*)

With additional investment from the United States Agency for International Development, the European Union and the Government of Japan, WHO was able to expand national EMT development support to additional PICs beginning in 2019, including Kiribati, the Marshall Islands, the Federated States of Micronesia (FSM), Palau, Papua New Guinea, Samoa and Tuvalu. (*4*, *5*, *9*) While national borders remained closed in multiple PICs during 2020–2022 due to the COVID-19 pandemic, online training was provided to Pacific EMTs through an 11-week webinar series in 2021, hosted by WHO and with faculty and participants from across the Pacific. (*9*)

In-person EMT training workshops and simulation exercises based on the WHO Blue Book recommenced in mid-2022, with team member trainings held in Fiji, Kiribati, the Marshall Islands, Palau and Samoa using the tailored Pacific EMT training package. (*10*) These trainings marked the formal launch of several EMTs, including the Kiribati Medical Assistance Team (KIRIMAT), the Marshall Islands Medical Assistance Team (MIMAT), Palau’s KLEMAT, and the Samoa Emergency Medical Assistance Team (SEMAT). (*5*) At the same time, with support from multiple donor partners, WHO undertook large-scale international procurement of curated cache kits designed specifically for lightweight, mobile Pacific EMTs to ensure that all teams are fully equipped and prepared for self-sufficient deployments. (*11*)

Pacific EMTs are considered “Type 1” according to standards set out in the WHO Blue Book. They are either fixed or mobile and are capable of providing emergency and outpatient care during daylight hours. The Fiji Emergency Medical Assistance Team (FEMAT) has also developed deployable surgical capacity. Pacific EMTs vary in size and composition, primarily based on human resource limitations in their countries. In Fiji, FEMAT now has a roster of over 500 potential members, of whom approximately 70 are trained, while teams in smaller PICs may have only 30 team members trained, with the capability to deploy 6–10 members at a time without undermining routine health services. Pacific EMT members include physicians, nurses, allied health professionals, public health experts, environmental health specialists, logisticians, firefighters, police and military, depending on national arrangements in each country.

## ACCOMPLISHMENTS OF PACIFIC EMTs

Pacific EMTs have made substantial progress since they started developing in 2017.

In 2019, FEMAT became the first Pacific EMT to achieve international classification. FEMAT was activated and deployed for seven national emergencies between 2019 and 2021, including multiple tropical cyclones and outbreaks, and was heavily involved in the country’s COVID-19 response. (*6*, *7*) FEMAT undertook its first international deployment to Tuvalu in 2022 to support its neighbour’s COVID-19 response, and deployed to Vanuatu in 2023 to support the response to back-to-back Tropical Cyclones Judy and Kevin.
The Solomon Islands Medical Assistance Team (SOLMAT) deployed alongside 17 other international EMTs to Samoa in 2019 in response to a massive measles outbreak. The team also supported national COVID-19 response efforts in 2022, among other national deployments.
In 2022, the Tonga Emergency Medical Assistance Team (TEMAT) responded independently to the Hunga-Tonga Hunga-Ha’apai volcanic eruption and subsequent tsunami while the country’s borders were closed. (*8*) TEMAT deployed to the Ha’apai islands for over 6 weeks, caring for nearly 400 patients affected by the eruption and tsunami.
The Vanuatu Medical Assistance Team (VANMAT) responded independently to the category 5 Tropical Cyclone Harold in 2020, when COVID-19 travel restrictions prevented deployment of international responders, and was activated in response to Tropical Cyclones Judy and Kevin in 2023.

## Discussion

Pacific EMTs have become critical resources for emergencies, and are supporting national responses to outbreaks and disasters, often without reliance on international EMTs for the provision of clinical services. The progress made since 2017 has demonstrated that even small and developing countries can develop and mobilize professional EMTs capable of timely, self-sufficient and high-quality responses to a wide range of emergencies, highlighting the importance of this capability for rapid response, and the significant return on investment when emergencies strike.

Looking forward, there are opportunities for Pacific EMTs to continue to improve and develop. Training for Pacific EMTs continues to evolve, based on the Blue Book standards and practical lessons from deployments. As expertise grows within PICs, national EMT leaders and team members can become faculty for peer EMT training, both in their own countries and internationally. Documented evidence on EMT operations and their effectiveness remains limited, presenting opportunities for Pacific EMTs to conduct operational research and evaluations of their deployments, and to measure and report on their impact in emergency settings.
